# Habitat fragmentation is associated with dietary shifts and microbiota variability in common vampire bats

**DOI:** 10.1002/ece3.5228

**Published:** 2019-05-09

**Authors:** Melissa R. Ingala, Daniel J. Becker, Jacob Bak Holm, Karsten Kristiansen, Nancy B. Simmons

**Affiliations:** ^1^ Richard Gilder Graduate School American Museum of Natural History New York New York; ^2^ Division of Vertebrate Zoology, Department of Mammalogy American Museum of Natural History New York New York; ^3^ Odum School of Ecology University of Georgia Athens Georgia; ^4^ Center for the Ecology of Infectious Disease University of Georgia Athens Georgia; ^5^ Department of Biology Indiana University Bloomington Indiana; ^6^ Department of Biology University of Copenhagen Copenhagen Denmark; ^7^ Clinical‐Microbiomics Copenhagen Denmark; ^8^ BGI Shenzhen China

**Keywords:** *Desmodus rotundus*, diet homogenization, land‐use change, livestock, microbiota, resource provisioning

## Abstract

Host ecological factors and external environmental factors are known to influence the structure of gut microbial communities, but few studies have examined the impacts of environmental changes on microbiotas in free‐ranging animals. Rapid land‐use change has the potential to shift gut microbial communities in wildlife through exposure to novel bacteria and/or by changing the availability or quality of local food resources. The consequences of such changes to host health and fitness remain unknown and may have important implications for pathogen spillover between humans and wildlife. To better understand the consequences of land‐use change on wildlife microbiotas, we analyzed long‐term dietary trends, gut microbiota composition, and innate immune function in common vampire bats (*Desmodus rotundus*) in two nearby sites in Belize that vary in landscape structure. We found that vampire bats living in a small forest fragment had more homogenous diets indicative of feeding on livestock and shifts in microbiota heterogeneity, but not overall composition, compared to those living in an intact forest reserve. We also found that irrespective of sampling site, vampire bats which consumed relatively more livestock showed shifts in some core bacteria compared with vampire bats which consumed relatively less livestock. The relative abundance of some core microbiota members was associated with innate immune function, suggesting that future research should consider the role of the host microbiota in immune defense and its relationship to zoonotic infection dynamics. We suggest that subsequent homogenization of diet and habitat loss through livestock rearing in the Neotropics may lead to disruption to the microbiota that could have downstream impacts on host immunity and cross‐species pathogen transmission.

## 1 INTRODUCTION

The animal gut microbiota plays an essential role in maintaining host health, including modulating effects of nutrition and immunity (Amato et al., 2014; Hanning & Diaz‐Sanchez, 2015; Khosravi & Mazmanian, 2013; O'Sullivan et al., 2013). However, the microbiota is not a static entity, and community composition can change rapidly in response to shifts in host diet (David et al., 2014; Turnbaugh, Ridaura, Faith, Rey, & Gordon, 2009). If such shifts lead to functional aberrations in community membership or composition—a pathological state called “dysbiosis”—nutritional fitness and host capacity to resist infection may be reduced (Khosravi & Mazmanian, 2013; Stecher, Maier, & Hardt, 2013; Williams et al., 2016). A primary mechanism by which gut microbial communities can influence infection is by altering the maintenance or development of the host immune system; bacteria associated with the gut epithelium can produce ligands that interact with toll‐like receptors in host cells, stimulating immune response cascades (Hooper, Littman, & Macpherson, 2012; Kau, Ahern, Griffin, Goodman, & Gordon, 2011; Macpherson & Harris, 2004; O'Sullivan et al., 2013; Thaiss, Zmora, Levy, & Elinav, 2016). For example, laboratory studies of mice illustrate that commensal microbiota contribute to the host's ability to mount proper immune responses (e.g., adaptive immunity) against viral infection (Ichinohe et al., 2011). Similarly, administration of probiotic bacteria to brown trout (*Salmo trutta*) increases the activity of the complement system, also suggesting links between the microbiota and innate immune function in animals (Balcázar et al., 2007). Compared to studies of laboratory animals and humans, interactions between endosymbiotic microbial communities and the host immune system are less well understood for wildlife (Evans, Buchanan, Griffith, Klasing, & Addison, 2017; Pedersen & Babayan, 2011), which are regularly exposed to pathogens and to environmental variation (McKenzie et al., 2017).

Environmental variation can be stark when humans rapidly alter the landscape for other uses. A growing body of research suggests that animal microbiotas may respond to land‐use change, particularly where such change leads to altered or deficient food resources. For example, black howler monkeys (*Alouatta pigra*) from fragmented forests display low dietary diversity that is associated with less diverse microbiota (Amato et al., 2013). The gut microbiotas of Udzungwa red colobus monkeys (*Procolobus gordonorum*) in fragmented forests were found to have significantly lower microbiota alpha diversity than conspecifics living in undisturbed forests and have reduced functional capacity to digest toxic xenobiotics naturally present in their diet (Barelli et al., 2015). These studies suggest that land‐use change and habitat loss may impact the fitness of animal microbiotas through changes in host diet. However, these patterns are not evident for all host species, nor across all spatial scales. One study of a community of African primates found that gut microbiotas were most strongly structured by host species identity and were largely resistant to perturbation even across fragmentation gradients (Mccord et al., 2014). Significant associations between microbial community structure and land‐use change have also been reported for some select taxa (e.g., amphibians; Becker, Longo, Haddad, & Zamudio, 2017; Reyes et al., 2017), but for most host taxa, there is little understanding whether land‐use change influences the gut microbiota.

Even where evidence exists that habitat quality impacts microbiota structure, how such ecological variation in diversity and structure relates to host immune defense in natural systems remains largely unquantified (Woodhams et al., 2014) in spite of relevance for the monitoring and control of reservoir hosts for zoonotic pathogens (Altizer et al., 2018). From a conservation perspective, understanding the potentially powerful but indirect effects of habitat destruction on animal microbiotas may also play a critical role in host conservation and management (Trevelline, Fontaine, Hartup, & Kohl, 2019; Wei et al., 2018; West et al., 2019). Under the “One Health” concept, which recognizes that the health of wildlife is interdependent with the health of humans and livestock (Zinsstag, Schelling, Waltner‐Toews, & Tanner, 2011), microbiotas may dually serve to monitor host health and track potential emerging zoonoses.

**Figure 1:**
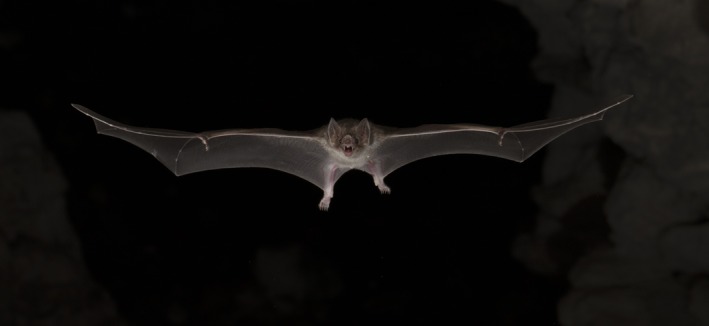
The common vampire bat, *Desmodus rotundus*. Photo credit: Brock and Sherri Fenton

In this study, we investigated the interplay among diet, microbiota structure, and innate immune function in a free‐ranging bat species, the common vampire bat (*Desmodus rotundus*, hereafter “vampire bats”) (Figure 1). Bats as a group harbor more zoonotic viruses than other mammalian orders and have been implicated in the spillover of pathogens such as Hendra virus, *Bartonella mayotimonensis*, and Marburg virus (Amman et al., 2012; Olival et al., 2017; Plowright et al., 2015; Veikkolainen, Vesterinen, Lilley, & Pulliainen, 2014). Vampire bats, in particular, are widely distributed from northern Mexico to northern Argentina and, owing to their blood‐feeding diet, can transmit pathogens such as rabies virus. Rabies is a major threat to livestock and human health in Latin America for which vampire bats are the main reservoir host (Greenhall & Schmidt, 1988; Schneider et al., 2009). Feeding on vertebrate blood may also facilitate cross‐species transmission of other pathogens such as *Bartonella,* hemoplasmas, influenza, and trypanosomes (Becker, Bergner, et al., 2018a; Hoare, 1965; Tong et al., 2013; Volokhov et al., 2017). Agricultural intensification in Latin America (i.e., the conversion of forests into livestock pasture) provides vampire bats with an abundant and accessible source of mammalian prey that has been implicated in shaping bat immune phenotypes. However, exactly how land conversion influences the vampire bat gut microbiota, and if this helps explain observed immune profiles, remains unknown (Altizer et al., 2018; Becker, Bergner, et al., 2018a; Streicker & Allgeier, 2016).

We approached these questions by sampling blood, rectal microbiota, and hair from vampire bats at two adjacent sites in Belize that contrast in land use. We first characterized the vampire bat gut microbiota using 16S amplicon sequencing of rectal swab samples. We chose this sampling scheme because previous studies have shown that rectally collected stool communities are more diverse than the intestinal lumen and record a strong signature of diet, one our principle drivers of interest (Araújo‐Pérez et al., 2012; Ingala, Simmons, Wultsch, & Krampis, 2018). Next, we measured long‐term diet using stable isotopes of carbon (δ^13^C) and nitrogen (δ^15^N) from hair. Vampire bats feeding primarily on livestock can be differentiated from those feeding on wildlife using δ^13^C, as most grasses consumed by livestock (e.g., cattle) use the C4 photosynthetic pathway while most forest plants consumed by forest wildlife (e.g., peccary) use the C3 pathway (Streicker & Allgeier, 2016; Voigt & Kelm, 2006a). δ^15^N also provides inference into the trophic level of prey species, as consumer δ^15^N is enriched by 3‰−4‰ relative to its diet (Post, 2002). We next tested if bat sampling site and long‐term diet (derived from hair samples) predict gut microbial diversity and composition. Lastly, we assessed whether microbiota attributes were associated with a functional measure of innate immune defense in the bats using plasma. Because vampire bats with access to domestic animals feed primarily on these prey (Streicker & Allgeier, 2016), we predicted that bats residing in a more agriculturally intensified site would show evidence of a more homogenous diet and have a gut microbiota with decreased alpha diversity (Becker, Czirják, et al., 2018b; Streicker & Allgeier, 2016; Voigt & Kelm, 2006a). We next predicted that less diverse vampire bat microbiotas would be associated with weaker immune defenses as measure by a bacterial killing assay (Khosravi & Mazmanian, 2013).

## 2 MATERIALS AND METHODS

### 2.1 Capture and sampling of vampire bats

**Figure 2:**
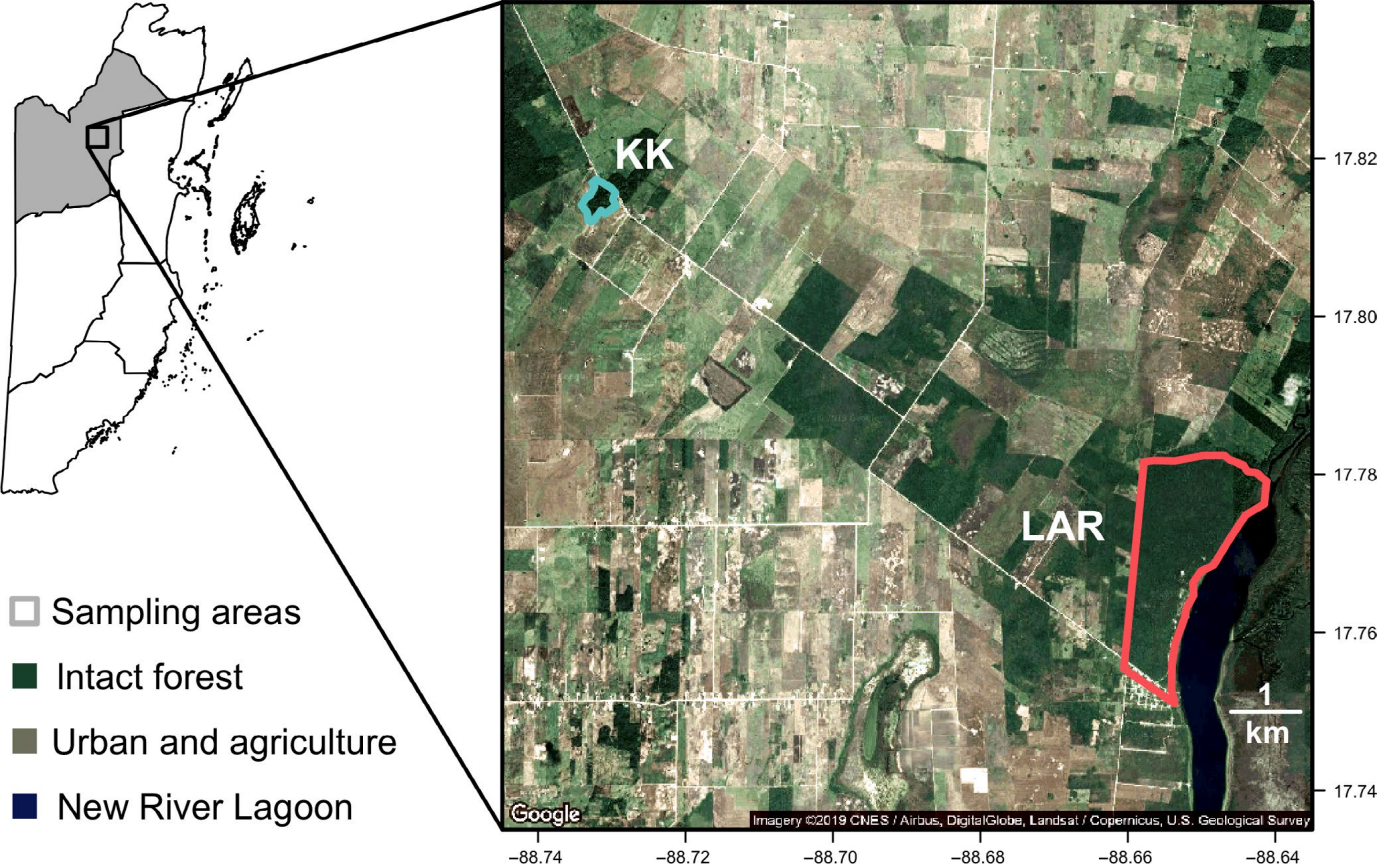
Detailed map of study sites in northern Belize. The shaded inset shows the location of Orange Walk District. Colored borders show the bounds of Lamanai Archaeological Reserve (LAR) and Ka'Kabish (KK). Prior to 2015, these sites were connected by contiguous forest (green), but now are disconnected by agricultural matrix (white and tan). Satellite imagery was derived from Google Maps, retrieved March 2019

Between April 20 and 25, 2015, we sampled 36 vampire bats from two adjacent areas in the Orange Walk District of Belize: Lamanai Archaeological Reserve (LAR) and Ka'Kabish (KK). LAR is a protected area bordered by the New River Lagoon, forest, and agricultural habitat, whereas KK is a site of remnant forest surrounded by clear‐cut agricultural fields (Herrera, Duncan, Clare, Fenton, & Simmons, 2018). The forest at LAR consists largely of closed‐canopy semi‐deciduous forest interspersed with areas of old secondary growth located within several kilometers of cattle pastures. While the forest at LAR is of higher quality than at KK, its proximity to cattle pastures still falls within the home range area for *D. rotundus* (Trajano, 1996). At KK, the forest is ecologically similar but more degraded, with smaller trees, a lower canopy, and more secondary vegetation. The area sampled at KK was limited entirely to forest regrown on Maya structures and plazas. Sampling sites were 8 km apart and consisted of roosts in a hollow tree and a colonial cistern (LAR) and looter's tunnels in an unexcavated Maya temple complex (KK) (Figure 2). Bats were captured with mist nets at roost entrances or along nearby flight paths from 19:00 until 22:00; a harp trap was also set from 18:00 to 05:00. Upon capture, bats were placed in individual cloth bags and issued a uniquely coded wing band (3.5 mm; Porzana Inc.).

For analyses of the vampire bat gut microbiota, we collected rectal samples from 30 bats (*n*_LAR_ =* *14, *n*_KK_ = 16) using sterile miniature rayon swabs (1.98 mm; Puritan). Rectal samples were flash‐frozen in liquid nitrogen for optimal bacterial DNA preservation (Hale, Tan, Knight, & Amato, 2015). For analyses of vampire bat diet, we trimmed <5 mg hair from the interscapular region of each individual for stable isotope analysis. To quantify innate immune defense, we obtained blood samples by lancing the propatagial vein with sterile 23‐gauge needles and collecting 20–50 μl of blood in heparinized capillary tubes. Plasma was isolated by centrifuging blood in serum separator tubes and was subsequently frozen at −20°C until transfer to −80°C storage at the University of Georgia. Following sampling, all bats were released at the sites where they were captured. We were unable to collect all sample types (hair, blood, rectal swabs) for all bats, and sample sizes vary (swabs = 30 individuals, hair = 29, swabs and hair = 23, blood = 20).

All field procedures followed guidelines of the American Society of Mammalogists (Sikes et al., 2016) and were approved by the University of Georgia Animal Care and Use Committee (AUP A2014 04‐016‐Y3‐A5). Fieldwork was approved by the Belize Forestry Department under permit CD/60/3/15(21).

### 2.2 Microbiota diversity and community composition

Host and microbial DNA was extracted using Macherey–Nagel Nucleospin Soil kit according to the manufacturers’ protocol (Macherey‐Nagel, Inc.). PCR‐based library formation targeting the 16S rRNA gene variable region 4 (V4) was performed using forward primer 515F (5′ AATGATACGGCGACCACCGAGATCTACAC NNNNNNNN TATGGTAATTGTGTGCCAGCMGCCGCGGTAA 3′, where “N” indicates the nucleotides of the barcode sequence) and reverse primer 806R (5′ CAAGCAGAAGACGGCATACGAGAT NNNNNNNNNNNN AGTCAGTCAGCCGGACTACHVGGGTWTCTAAT 3′) with Illumina adaptor sequences on the 5′ end (Apprill, Mcnally, Parsons, & Weber, 2015; Caporaso et al., 2011). Prepared libraries were sequenced on the Illumina MiSeq platform using 2 × 350 bp v3 chemistry.

**Figure A1:**
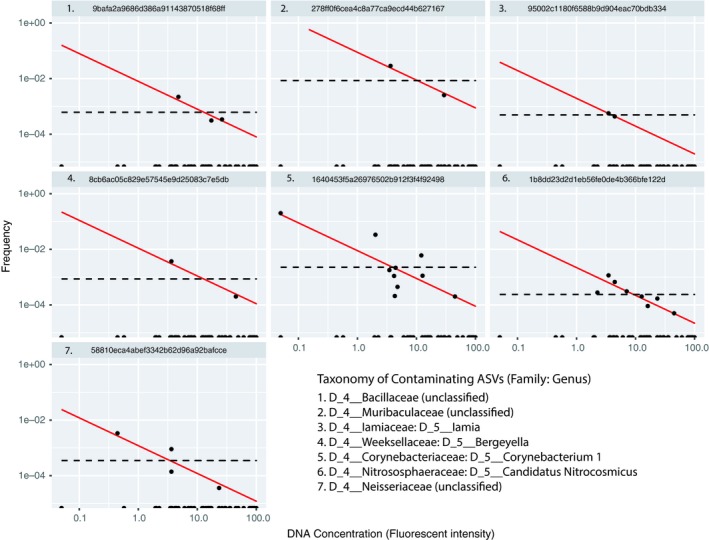
Contaminating ASVs identified by *decontam* frequency filtering. *X*‐axis shows the initial DNA concentration of microbiome samples as measured by fluorescent quantitation. The *Y*‐axis shows the frequency of the sequence variant. All ASVs shown above fit the contaminant model with *p* < 0.05

We recovered an average raw read depth of 40,945 sequences per sample (*SE*_mean_ = 3,160). Raw reads were processed using QIIME2 v. 2019.1 (Bolyen et al., 2018). We used the DADA2 QIIME2 plugin to denoise and quality filter reads, call amplicon sequence variants (ASVs), and generate a feature table of ASV counts and host metadata (Callahan et al., 2016). After quality filtering with DADA2, the average read depth across samples was 28,415 (*SE*_mean_ = 2,345). We assigned bacterial taxonomy to the ASV feature table using the Naive Bayesian Q2 feature classifier as implemented in QIIME2, comparing against a SILVA reference database trained on the 515F/806R region of the 16S gene (Bokulich et al., 2018; Karst et al., 2016). Next, we frequency‐filtered potential contaminants from the ASV feature table using the initial sample DNA concentrations and R package *decontam* (*v.1.2.1*) (Figure A1A1; Davis, Proctor, Holmes, Relman, & Callahan, 2018). To determine the core set of ASVs characterizing the vampire bat microbiome, we used the core_members function from the *microbiome* R package (http://microbiome.github.io), setting the prevalence threshold at 50% (Caporaso et al., 2010). Prior to statistical analysis of microbiota, we produced rarefaction curves in QIIME2 in order to choose an appropriate minimum rarefying depth (*n* = 10,000).

### 2.3 Quantifying vampire bat diet

We used previously published data on stable isotopes of carbon (δ^13^C) and nitrogen (δ^15^N) from hair samples (*n*_total_ = 29; *n*_LAR_ = 13, *n*_KK_ = 16). to infer long‐term feeding patterns of each individual bat (Becker, Czirják, et al., 2018b). δ^13^C and δ^15^N were quantified from dried fur with a Thermo Delta V isotope ratio mass spectrometer at the University of Georgia Center for Applied Isotope Studies.

### 2.4 Measuring functional immune defense

Innate immune function data for this study were published previously (Becker, Czirják, et al., 2018b); briefly, we assessed a functional measure of the innate immune defense in individual vampire bats by quantifying the ex vivo bacterial killing ability (BKA) of plasma samples against *Escherichia coli* ATCC 8739 (Tieleman, Williams, Ricklefs, & Klasing, 2005). In bat plasma, this pathogen is cleared mainly through complement proteins (Moore et al., 2011). Using the methods described in Becker, Chumchal, et al. (2017), we used the microplate reader method with 1:8 dilutions of plasma to phosphate‐buffered saline run in duplicate and challenged with a 10^4^ bacteria/ml solution (E power Microorganisms #0483E7; Microbiologics Inc). BKA is thus expressed at the percentage of *E. coli* cleared by the sample relative to the positive control.

### 2.5 Statistical analyses

We first used linear regressions to assess if δ^13^C and δ^15^N varied per site. We also derived the standard ellipse area corrected for small sample size (SEAc) for δ^13^C and δ^15^N as a proxy for the diversity of feeding strategies and thus dietary homogenization per site (Jackson, Inger, Parnell, & Bearhop, 2011). We used a permutational multivariate analysis of variance (PERMANOVA) to assess differences in isotopic position (matrix of δ^13^C and δ^15^N) according to site.

We next assessed whether site and long‐term bat diet (δ^13^C and δ^15^N) predicted microbiota diversity using univariate and multivariate tests. For alpha diversity, we fit linear regression models with log‐transformed Shannon diversity as the dependent variable and separately included site (*n* = 30) and both δ^13^C and δ^15^N (*n* = 23) as predictors; site and diet were not included in the same model owing to the different sample size and to avoid overfitting. To test for differences in microbial beta diversity according to site and diet, we performed a PERMANOVA on both weighted and unweighted UniFrac distances using the *vegan (v. 2.5‐4)* package (Lozupone, Hamady, & Knight, 2006; Oksansen et al., 2017). Mantel tests were performed to test for associations between δ^13^C/δ^15^N distance matrices and both weighted and unweighted Unifrac matrices. We visualized differences in microbial membership and composition according to sampling site using principal coordinates analysis (PCoA) using package *phyloseq (v. 1.26.1)* (Gower, 1966; McMurdie & Holmes, 2013).

We used *DESeq2* (*v. 1.22.2*) (Love, Huber, & Anders, 2014; McMurdie & Holmes, 2014) to test the effects of diet (as inferred by δ^13^C and δ^15^N) on microbiota composition on an ASV‐by‐ASV basis. *DESeq2* models raw OTU counts using a negative binomial distribution that accounts for differences in library sizes and determines significance of explanatory variables using the Wald test (Love et al., 2014). Using this model, we tested for ASV enrichment as a function of binned δ^13^C values. Following previous studies of vampire bats, we considered values within the interval of −13 to −7 to be representative of more‐livestock diets, with values outside this interval corresponding to less‐livestock diets (Voigt & Kelm, 2006a). We considered ASVs to be significantly enriched if the Benjamini–Hochberg adjusted *p* value was <0.05.

Finally, we assessed the relationship between BKA and (a) the relative abundance of core ASVs identified above and (b) alpha diversity to test how the community composition and diversity of the gut microbiota correlate with innate immune defense. We transformed proportional BKA to be bound within the 0 and 1 interval and used the logit‐transformed values as the dependent variable in a series of robust linear mixed effects models with the *robustlmm* package (Koller, 2016). Robust estimation methods help minimize the effect of potential outliers, which is particularly helpful when sample sizes are small. We modeled separate univariate relationships between microbial data (we used a square‐root transformation for the relative abundance of each core ASV; Zuur, Ieno, & Elphick, 2009) and BKA and included assay plate as a random effect. As rlmer() does not report *p* values, we report the slope and 95% confidence interval for each predictor (e.g., each core relative abundance and alpha diversity), interpreting no overlap with zero as statistically significant.

## 3 RESULTS

### 3.1 Dietary differences between sites

**Figure 3:**
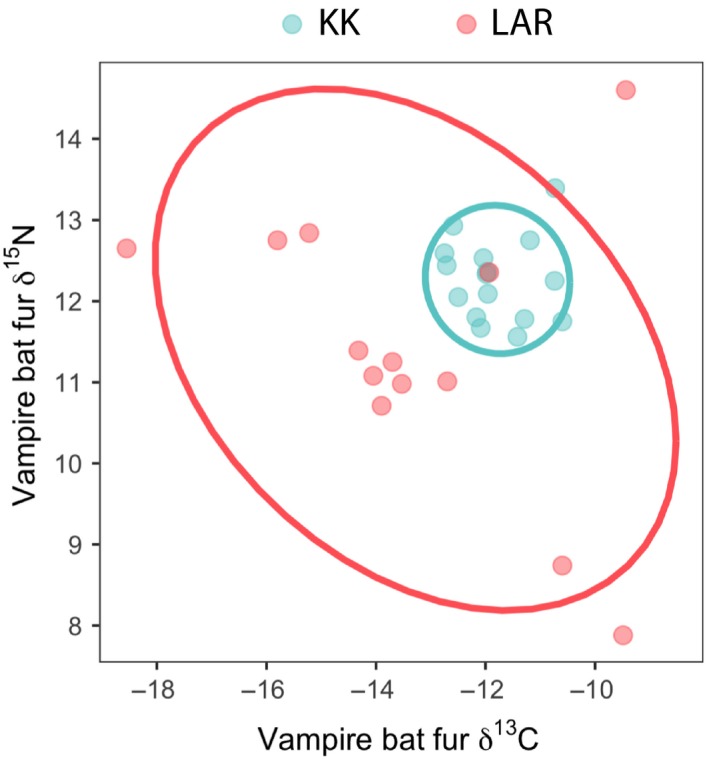
Site variability in stable isotopes of carbon (δ^13^C) and nitrogen (δ^15^N) from vampire bats sampled in Belize (*n *= 29). Points show individual diet data from all bats sampled in 2015, while lines show the standard ellipse corrected for small sample size (SEAc). Red points = LAR (*n* = 13), blue points = Ka'Kabish (*n* = 16)

Within our sample of 29 vampire bats for which stable isotope data were available, individuals foraging within LAR (intact) and KK (fragmented) varied by δ^13^C (*β*_LAR_ = −1.53, *t* = −2.29, *p* = 0.03) but only marginally by δ^15^N (*β*_LAR_ = −0.86, *t* = −1.89, *p* = 0.07). However, KK bats showed narrower isotopic niche width (SEAc = 1.21) compared to LAR bats (SEAc = 14.41; Figure 3). Accordingly, the PERMANOVA showed that site predicted bat isotopic position (*F*_1,27_ = 4.7, *R* ^2^ = 0.15, *p* = 0.01).

### 3.2 Core microbiota summary

**Figure 4:**
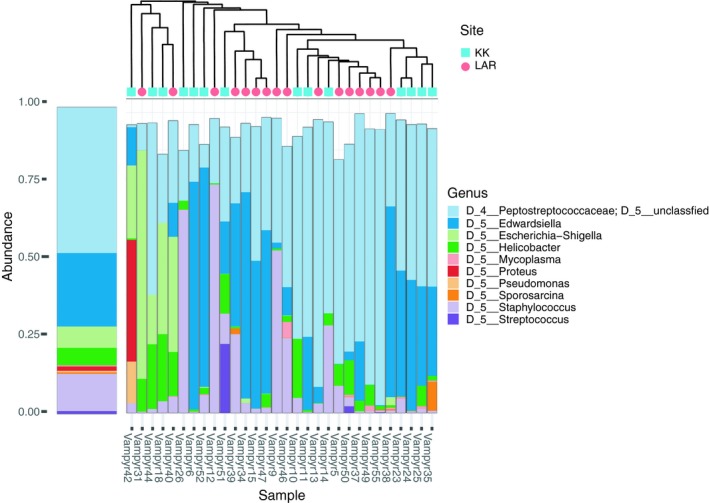
Composition of vampire bat microbiotas (*n* = 30), showing relative abundance of top 25 bacteria at the genus level. Each stacked bar represents an individual bat. The dendrogram illustrates the similarity between the samples based on Bray–Curtis distance using the mean linkage method. The left bar illustrates relative abundance of bacterial genera across all samples

Contamination filtering identified a total of seven putative contaminants which were filtered from the feature table (Figure A1A1), and the resulting cleaned feature table was used for the core microbiota summary and all statistical analyses. The overall structure of the 30 vampire bat microbiotas revealed that the dominant bacterial families were the Peptostreptococcaceae (Phylum Firmicutes), Enterobacteriaceae (Phylum Proteobacteria), Helicobacteraceae (Phylum Epsilonbacteraeota), Staphylococcaceae (Phylum Firmicutes), Mycoplasmataceae (Phylum Tenericutes), Halomonadaceae (Phylum Proteobacteria), and Bacillaceae (Phylum Firmicutes) (Figure 4; Table 1). The core microbiome, defined as those ASVs represented in >50% of the 30 samples (rarefied to 10,000 reads per sample), contained 12 ASVs (Table 1). Two ASVs, an unidentified member of the family Peptostreptococcaceae and a member of the genus *Helicobacter*, were detected in all samples (Table 1). The average relative abundance of the Peptostreptococcaceae core ASV was 45.0%, whereas the average relative abundance of *Helicobacter* across samples was 4.5%.

**Table 1:** Amplicon sequence variants (ASVs) defining the core microbiota of Belizean vampire bats. To be considered part of the “core” microbiota, ASVs had to be present in >50% of the samples

ASV ID	Phylum	Family	Genus	Prevalence	Prevalence (%)	Avg. abundance (%)
fcd55911156149fdda9de94a733cc51c	Epsilonbacteraeota	*Helicobacteraceae*	*Helicobacter*	25	83.3	1.2
470e48a9a6ed554db28a235a56d894d2	Epsilonbacteraeota	*Helicobacteraceae*	*Helicobacter*	30	100	4.5
c17deeb81d27df533b8c5d09f80f672f	Proteobacteria	*Enterobacteriaceae*	*Edwardsiella*	21	70	13.9
50d98760f933669facdf7e0f5f4c7d2b	Proteobacteria	*Halomonadaceae*	*Halomonas*	26	86.7	0.2
952668c4024daf247606aa20c6f6d96e	Firmicutes	*Bacillaceae*	*Aeribacillus*	20	66.7	0.08
aa9d7191529c931ebd368615d0531c8a	Firmicutes	*Staphylococcaceae*	*Staphylococcus*	9	30	1.8
15d888a8d8e6c158974710b615dcbcdf	Firmicutes	*Staphylococcaceae*	*Staphylococcus*	21	70	3.5
56b584fbd736b3d1f678e06b8a059663	Tenericutes	*Mycoplasmataceae*	*Mycoplasma*	16	53.3	0.42
da16e0be25b62f12a3d9750211d42bd4	Tenericutes	*Mycoplasmataceae*	*Mycoplasma*	16	53.3	0.09
e261c20b5c702a2249153b9f5d1eac42	Firmicutes	*Peptostreptococcaceae*	*Unclassified*	16	53.3	0.53
c733ab7b076b07b518f42063ebc3351a	Firmicutes	*Peptostreptococcaceae*	*Unclassified*	30	100	45
993f939073eee4a78f5e61cc57784442	Firmicutes	*Peptostreptococcaceae*	*Unclassified*	16	53.3	0.32

### 3.3 Effects of site and diet on microbiome communities

**Figure 5:**
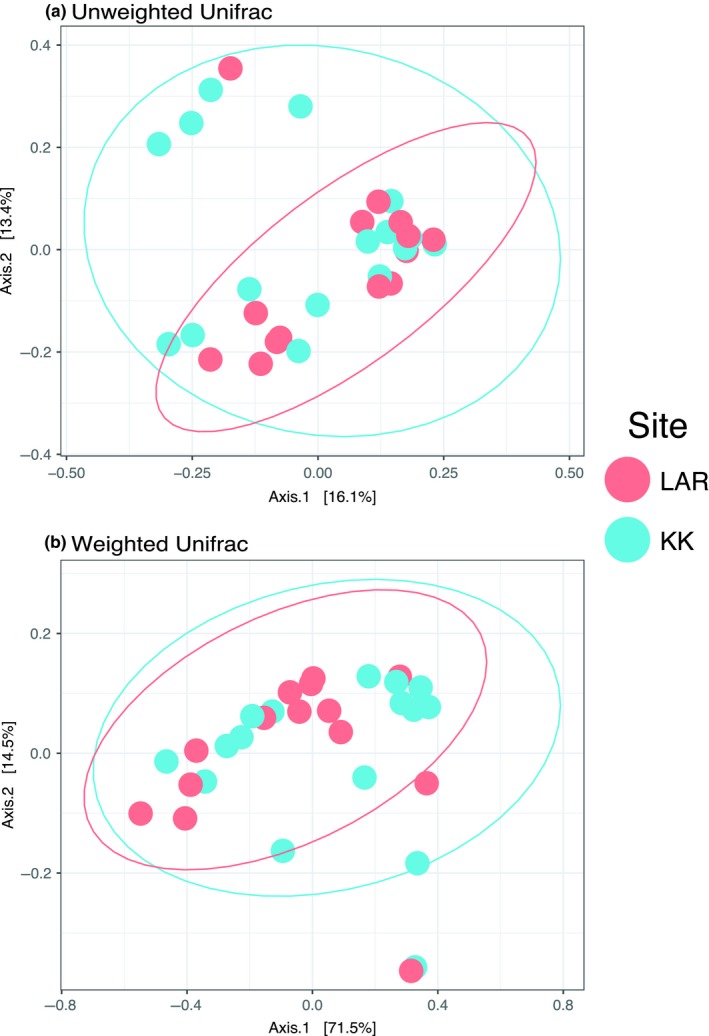
Principal coordinates analysis (PCoA) plots of vampire bat microbiotas sampled in Belize (2015) according to sampling site. Points represent (a) unweighted and (b) weighted UniFrac distances for individual microbiotas, while lines indicate 95% confidence ellipses around group centroid

Alpha diversity was not significantly different between sites (*F*_1,28_ = 1.6, *R* ^2^ = 0.06, *p* = 0.21), although LAR had weakly higher richness than KK (*β* = 0.15, *t* = 1.28). Diet variation did not predict alpha diversity through either δ^13^C (*β* = 0.05, *t* = 0.99, *p* = 0.33) or δ^15^N (*β* = 0.01, *t* = 0.22, *p* = 0.83). We found no statistically significant differences in bat gut microbial beta diversity between sites, as inferred by PERMANOVA on both unweighted (Figure 5; *F*_1,28_ **=* *1.13, *R* ^2^ = 0.039, *p* = 0.27) and weighted (Figure 5; *F*_1,28_ = 1.01, *R* ^2^ = 0.035, *p* = 0.34) Unifrac distances. However, multivariate dispersion around the centroid for unweighted Unifrac distances was significantly different between sites, indicating higher variability in community membership in KK compared with LAR (Figure 5; *F*_1,28_ = 5.0344, *N*_perm_ = 999, *p* = 0.04). Similarly, beta diversity of bat gut microbiotas did not differ as a function of δ^13^C (Mantel test, unweighted Unifrac *r* = −0.27, *p* = 0. 99; weighted Unifrac *r* = 0.05, *p* = 0.29) or δ^15^N (Mantel test, unweighted Unifrac, *r* = −0.29, *p* = 0.99; weighted Unifrac *r* = 0.10, *p* = 0.20).

**Figure 6:**
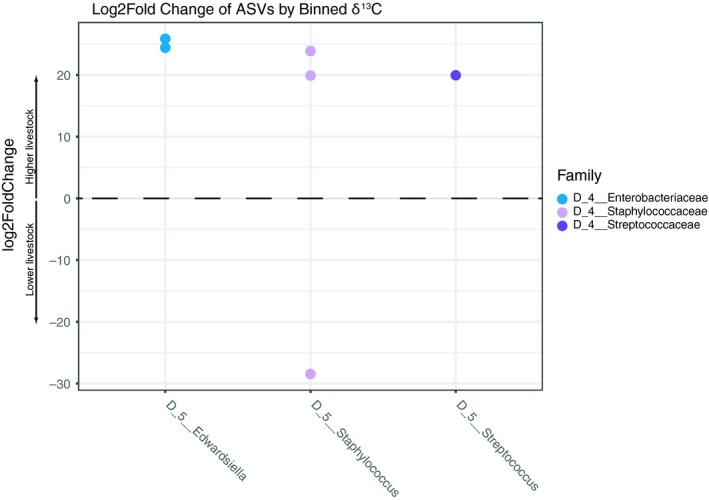
Significantly enriched ASVs stratified by low‐ or high‐livestock δ^13^C values. Points correspond to log2fold enrichment values, where values >0 reflect enrichment of the taxon in the high‐livestock bin compared to low‐livestock bin. Values <0 reflect enrichment of the taxon in the low livestock bin compared to the high livestock bin

**Figure B1:**
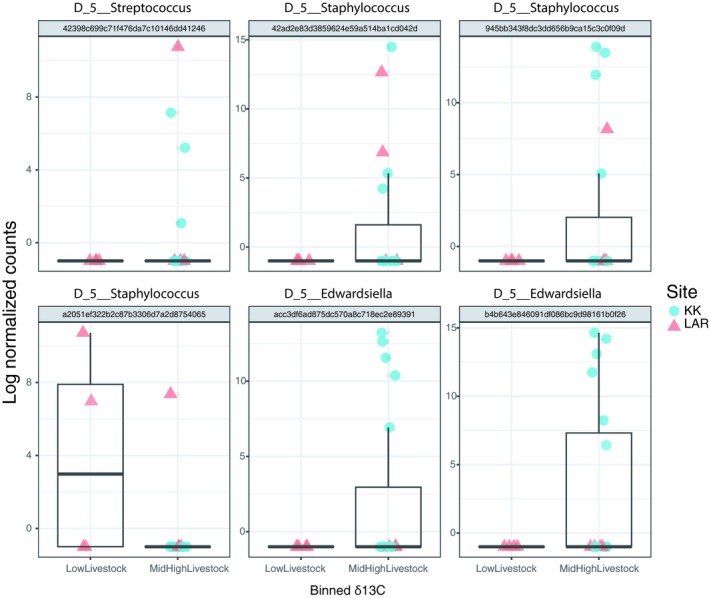
Log normalized counts of ASVs identified as differentially enriched between the low and high livestock δ^13^C bins (*p*_adj_ < 0.05). Points correspond to vampire bat individuals. *X*‐axis depicts whether that sample was included in the high‐livestock interval or the low‐livestock interval. Boxplots depict the median count bounded by lower and upper quartiles. The whiskers represent the standard error of the median values

To assess whether individual ASVs vary in response to diet, we performed an enrichment test using the *DESeq2* function. Because δ^13^C, but not δ^15^N, differed significantly between KK and LAR vampire bats, we performed enrichment analysis on δ^13^C values only. Analysis of binned δ^13^C values revealed that several core taxa belonging to the genera *Edwardsiella*, *Streptococcus*, and *Staphylococcus* were enriched in the high‐livestock group compared with the low‐livestock group (Figure 6, Table 2). Vampire bats from both sites fell across both δ^13^C bins (Figure B1B1), suggesting a decoupling of livestock consumption and sampling site. One ASV of the genus *Staphylococcus* was depleted in the high‐livestock group compared with the low‐livestock group (Figure 6).

### 3.4 Microbiota composition and innate immunity

**Figure 7:**
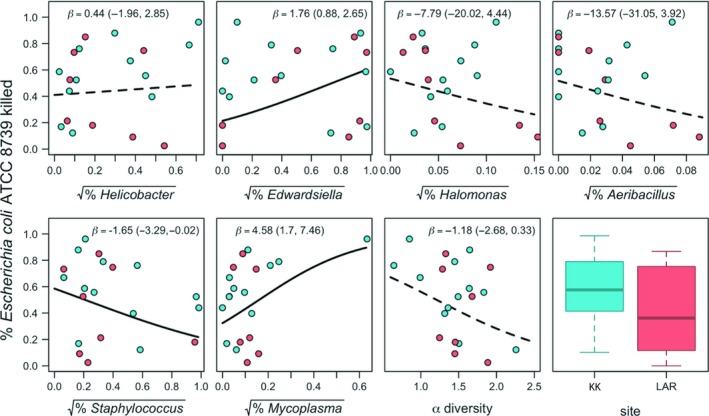
Robust LMMs testing the relationship between innate immunity and relative abundance of core gut microbiome taxa. Innate immunity was measured as % *Escherichia coli* killed during the assay by vampire bat blood serum. Hashed lines represent relationships where the 95% confidence interval overlaps with 0. Boxplot depicts Shannon diversity by site

The relative abundance of core ASVs was significantly associated with bat immune defense (i.e., BKA, *n*_total_ = 20; *n*_LAR_ = 8*, n*_KK_ = 12), with the direction and strength of this relationship differing among individual core ASVs (Figure 7). After accounting for outliers and assay plate, the relative abundances of *Edwardsiella* and *Mycoplasma* were both positively associated with BKA (*Edwardsiella*: *β* = 1.76, 95% CI = 0.88–2.65; *Mycoplasma*: *β* = 4.58, 95% CI = 1.7–7.46), while the relative abundance of *Staphylococcus* was negatively associated with BKA (*β* = −1.65, 95% CI = −3.29 to −0.02). The relative abundances of *Helicobacter*, *Halomonas*, and *Aeribacillus* showed no relationship with BKA (Figure 7). Our data suggested a trend for BKA to be negatively associated with alpha diversity, but confidence intervals overlapped with zero (*β* = −1.18, 95% CI = −2.68 to 0.33) .

**Table 2:** Differentially enriched amplicon sequence variants (ASVs) binned by low‐ or high‐livestock δ^13^C values

ASV ID	baseMean	log2FoldChange	padj	Phylum	Family	Genus
b4b643e846091df086bc9d98161b0f26	551.468504	25.86	5.96E‐09	Proteobacteria	Enterobacteriaceae	*Edwardsiella*
acc3df6ad875dc570a8c718ec2e89391	193.62763	24.41	4.40E‐08	Proteobacteria	Enterobacteriaceae	*Edwardsiella*
42ad2e83d3859624e59a514ba1cd042d	7.55500713	19.90	1.69E‐05	Firmicutes	Staphylococcaceae	*Staphylococcus*
a2051ef322b2c87b3306d7a2d8754065	78.719487	−28.46	2.66E‐11	Firmicutes	Staphylococcaceae	*Staphylococcus*
945bb343f8dc3dd656b9ca15c3c0f09d	185.036165	23.88	7.69E‐08	Firmicutes	Staphylococcaceae	*Staphylococcus*
42398c699c71f476da7c10146dd41246	7.80057541	19.95	1.69E‐05	Firmicutes	Streptococcaceae	*Streptococcus*

Note

Positive log2fold change coefficients indicate that the ASV is more abundant in the high‐livestock group compared to low‐livestock group. Negative values indicate depletion of that ASV in the high‐livestock group compared to the low‐livestock group.

## 4 DISCUSSION

Common vampire bats (*D. rotundus*) are one of three species of vampire bats found in the Neotropics and are the most specialized for feeding on mammalian prey (Goodwin & Greenhall, 1961; Greenhall, Joermann, Schmidt, & Seidel, 1983; Greenhall & Schmidt, 1988; Greenhall, Schmidt, & Lopez‐Forment, 1969; Turner, 1975). As such, vampire bats are uniquely poised to experience diet‐induced shifts in physiology and microbiota structure as a result of livestock rearing in the Neotropics. We demonstrate here that land‐use change even at relatively fine spatial scales (*c*. 8 km) is associated with differences in vampire bat diets that may influence the abundance of core microbiome members. We found significant differences in diet breadth between vampire bats captured at the fragmented‐forest site (KK) versus the contiguous forest site (LAR) (Figure 3). Vampire bats from KK had δ^13^C signatures more indicative of consistent feeding on livestock (Becker, Chumchal, et al., 2017; Voigt & Kelm, 2006b) and narrower dietary breadth, suggesting greater dietary homogenization at KK than LAR, where diets were far more variable. This pattern of diet homogenization at KK is generally consistent across sampling years (2014–2017; D. J. Becker, unpublished data), suggesting that these data are representative of the long‐term trends in vampire diets at the two sites. Studies of stable isotopes and bloodmeal analysis suggest that vampire bats likely prefer cattle because cattle populations are continuously present and exist in higher density than native prey mammals (Becker, Czirják, et al., 2018b; Bobrowiec, Lemes, & Gribel, 2015; Voigt & Kelm, 2006b). Our study supports this diet preference and suggests that bats in protected, mature forests have more variable diets than those in isolated forest fragments adjacent to cattle pastures, even though some individuals from LAR had δ^13^C signatures which fell into the high‐livestock group (Figure B1B1). A possible explanation for higher diet variability at LAR is that bats in larger forest tracts must forage over wider areas because their prey is more widely dispersed across the landscape. However, more detailed studies of vampire bat movement using global positioning systems (GPS) radiotracking are needed to confirm this hypothesis (Fenton et al., 1992).

We report here a core vampire bat microbiota composed mostly of members of the phyla Firmicutes, Proteobacteria, Tenericutes, and Epsilonbacteraeota (Figure 3, Table 1). This composition is consistent with previous reports of vampire bat microbiotas (Carrillo‐Araujo et al., 2015; Ingala et al., 2018; Phillips et al., 2012). Vampire bats have gut microbiotas that are compositionally distinct from those of other bats, most likely as a result of selective pressures to optimize bacterial metabolism of bloodmeals (Zepeda Mendoza et al., 2018). Indeed, vampire bats share several key hemolytic bacterial symbionts, such as *Aeromonas* spp., with other blood‐feeding animals such as leeches (Muller, Pinus, & Schmidt, 1980), suggesting that hematophagous animal microbiome composition is closely associated with this specialized diet.

Given that diet is known to be a strong driver of microbiota community composition and function (Muegge et al., 2012; Phillips et al., 2017) and that some differences exist in vampire bat dietary breadth between KK and the LAR, we tested for differences in the microbiotas of vampire bats between these contrasting sites. We found no significant differences in either alpha or beta diversity between sites (Figure 4). Previous studies in primates have found that degraded habitats are associated with lower microbiota diversity at similar spatial scales (Amato et al., 2013; Barelli et al., 2015), yet we failed to detect a difference here. It is possible that vampire bats in this region of Belize are not philopatric to particular roosts, such that some of our captures at KK could have been previously foraging or living in LAR, and vice versa. In this scenario, we would expect any differences in microbiota structure or membership between sites to be confounded. More detailed studies on vampire bat roost fidelity in Belize are needed, as there appears to be variability in roost philopatry across the range of *D. rotundus*. For example, a study of Argentine vampire bats found that only about 15% of individuals were recaptured at the roost where they were initially captured (Delpietro, Russo, Carter, Lord, & Delpietro, 2017), while another study found no movement among Peruvian vampire bats in roosts located just 2.2 km apart (Streicker et al., 2012). Though we found no difference in beta diversity between sites, we did note increased heterogeneity of KK gut microbiotas compared with LAR, despite the fact that the bats at KK have more homogenous diets (Figure 3). Previous studies have reported similar patterns, where perturbed animal gut communities are characterized by increased dispersion around a “healthy” centroid rather than deterministic community shifts (Zaneveld, McMinds, & Thurber, 2017). We speculate that a possible explanation for this observation is that local livestock is treated with antibiotics which may be consumed by the vampire bats during feeding, triggering increased microbial turnover and influencing the heterogeneity in community structure we report here. More detailed data collection on local antibiotic use in livestock could be incorporated to test this hypothesis in the future.

Because we found differences in dietary breadth (Figure 3) but not microbiota structure (Figure 5) between KK and LAR, we also tested for correlations between diet and microbiota structure irrespective of sampling site; however, we also did not detect significant correlations between either weighted or unweighted Unifrac and δ^13^C distance matrices. We did not test for correlations between Unifrac matrices and δ^15^N, because we found no significant differences in this diet metric (which approximates trophic level) among our samples. Previous studies have demonstrated a very narrow range of δ^15^N in vampire bats from non‐coastal habitats (Streicker & Allgeier, 2016), and that wildlife and domestic prey do not vary appreciably in this isotope in this region of Belize (Becker, Longo, et al., 2017). Isotopic ratios measure long‐term dietary trends (Tieszen, Boutton, Tesdahl, & Slade, 1983) recorded on the time scale it takes the animal to generate the tissue (in our case, hair), whereas the gut microbial community can experience turnover within hours or days following a dietary shift (David et al., 2014). In light of this evidence, it is possible that stable isotopes of hair do not capture dietary changes on a time scale appropriate for analyzing links with microbial turnover in the gut. Future studies should consider using short‐term diet monitoring techniques, such as DNA metabarcoding of fecal samples (Clare et al., 2014), to better assess how microbiota composition changes in response to diet shifts.

Though overall community composition and membership were not related to long‐term diet, differential enrichment analysis showed that some individual core ASVs were enriched in the higher‐livestock group of bats compared to those with lower‐livestock diets (Figure 6). Importantly, both high and low δ^13^C values were distributed across vampire bats from the two sampling sites, indicating that bats in the contiguous forest site (LAR) are also leaving the reserve to forage on cattle (Figure S2). The reserve (LAR) is within several kilometers of cattle pastures (Figure 2), which falls within the reported home range size of 2–3 km^2^ for *D. rotundus* (Streicker et al., 2012; Trajano, 1996). Our results suggest that the interplay between changes in long‐term diet and shifts in microbiota are perhaps more complex than previously thought, while overall community structure may resist perturbation across sites, shifts in the relative abundance of select taxa such as those reported here could still have functional implications for host. Future studies could incorporate shotgun metagenomic techniques to more directly measure the functions associated with microbial taxa changing in response to diet.

Because vampire bats may be important reservoirs of zoonotic pathogens (Condori‐Condori, Streicker, Cabezas‐Sanchez, & Velasco‐Villa, 2013; Mayen, 2003; Volokhov et al., 2017; Zetun, Hoffmann, Silva, Souza, & Langoni, 2009) and the microbiota is known to modulate immunity (Khosravi & Mazmanian, 2013; Thaiss et al., 2016), we tested for associations between innate immune defense and microbiota structure. We found significant relationships between the proportional abundance of some core microbiota with the ability of host plasma to kill *E. coli* (Figure 7). Future studies that incorporate additional measures of bat immune phenotypes, especially those quantifying different arms of the immune system or expression of various immune genes (e.g., cytokines), could shed further light on links between the microbiota and infection dynamics (Demas, Zysling, Beechler, Muehlenbein, & French, 2011; Fassbinder‐Orth, 2014).

A limitation of amplicon‐based studies is that 16S regions often lack the sensitivity to distinguish among bacterial species and/or strains, which may have different metabolic functions despite sharing identical 16S sequences (Antony‐Babu et al., 2017). Future studies using shotgun metagenomic techniques could be used to analyze how the microbiota changes on a gene‐by‐gene basis in response to habitat quality, which may give more detailed insight to how these changes in vampire bats impact metagenome functions related to immunity. For example, a hologenomic study of vampire bats found that their microbiotas were characterized by an enrichment of potentially protective bacterial genes originating from *Amycolatopsis mediterranei*, which is known to produce antiviral compounds (Zepeda Mendoza et al., 2018). Expansion of our study (a) across a quantitative fragmentation gradient and (b) using shotgun metagenomic techniques could provide detailed insights into the functional consequences of habitat fragmentation on vampire bat microbiotas. In particular, the inclusion of a truly pristine forest site would better contextualize our isotopic data by providing a site where cattle are infrequently available or altogether absent.

In summary, we demonstrate here that changes in land use in Belize have measurable associations with both dietary breadth and gut microbiota variability, but not composition, in common vampire bats. We found bacterial genera respond to diet, but not site, suggesting that diet is related to but decoupled from sampling site in our dataset. Our results join a growing body of evidence suggesting that the microbiota can respond to environmental change. Even though we did not detect dysbiosis in these animals, we recognize that dysbiosis of the microbiota may be monitored as an early warning sign about potential downstream effects on host ecology and health (Bahrndorff, Alemu, Alemneh, & Lund Nielsen, 2016; Cheng et al., 2015; Lemieux‐Labonté, Simard, Willis, & Lapointe, 2017; Thomason, Mullen, Belden, May, & Hawley, 2017). We further showed that the relative abundances of some core microbiota members are related to innate immune function. While future immunological work could expand upon these findings and their implication for infection states, this pattern suggests that shifts in this subset of the microbiota could have especially profound impacts on host susceptibility to infections and on fitness. As agricultural land conversion continues in Belize (Cherrington et al., 2010), humans and their domestic animals will come into more frequent contact with wildlife, including vampire bats. It will be essential to continue monitoring the diets, habitat use, and microbiotas of vampire bats to test hypotheses about the impacts of these variables on zoonotic disease cycles, especially of rabies but also of other bacterial pathogens. Future studies integrating microbiota analysis, detailed dietary analysis, GPS radiotracking, and other tools can leverage the power of these techniques to expand on the foundational work we present here.

## 5 CONFLICT OF INTEREST

None declared.

## AUTHOR CONTRIBUTIONS

MRI analyzed and interpreted the data and wrote the manuscript. DJB collected field data, analyzed stable isotope and BKA data, secured funding, and helped write the manuscript. JBH collected rectal swabs, performed 16S amplicon sequencing, analyzed microbiota data, and helped write the manuscript. KK participated in discussions on metagenomics and analysis of 16S amplicon sequencing. NBS secured permits for field collection of samples, coordinated the field expedition, and helped write the manuscript.

## Data Availability

Raw, demultiplexed 16S sequence reads are archived publicly on the NCBI Short Read Archive (SRA) under BioProject #PRJNA506223. Sample metadata, ASV phylogeny, and taxonomically annotated ASV feature table are available on FigShare: https://figshare.com/projects/Habitat_Fragmentation_is_Associated_with_Dietary_and_Microbiome_Variability_in_Common_Vampire_Bats/57041.

## APPENDIX A

### CONTAMINATION ELIMINATION METHODS AND RESULTS

We eliminated potential contaminants using the *decontam* R package v. 1.2.1. Because we did not sequence a negative extraction control, we used *decontam*'s frequency‐filtering technique, which assumes a negative linear relationship between initial DNA concentration and frequency of the potential contaminant (Davis et al., 2018). If a contaminant model is a better fit to the data than a null model (where frequency is independent of DNA concentration), the amplicon sequence variant (ASV) is identified as a contaminant. The identified contaminants were removed from the *phyloseq* ASV feature table, and the cleaned feature table was used for all downstream analyses. The contaminants are shown in Figure A1A1.

## APPENDIX B

### DESEq2 ENRICHMENT RESULTS BY INDIVIDUAL AMPLICON SEQUENCE VARIANT

In the DESeq2 enrichment analysis, vampire bat samples from Ka'Kabish (KK) and Lamanai Archaeological Reserve (LAR) fell across both low‐ and high‐livestock bins, indicating that bats from these sites vary in the amount of livestock included in their diets. Of the six amplicon sequence variants (ASVs) found to be differentially enriched as a function of livestock consumption (Figure 6), nearly all had a mixture of individuals from KK and LAR (Figure B1B1).
