# Supplementary figures and images for: Habitat fragmentation is associated with dietary shifts and microbiota variability in common vampire bats
Source: Ecol Evol. 2019 May 9;9(11):6508–23. doi: 10.1002/ece3.5228 (PMC6580296; doi:10.1002/ece3.5228)

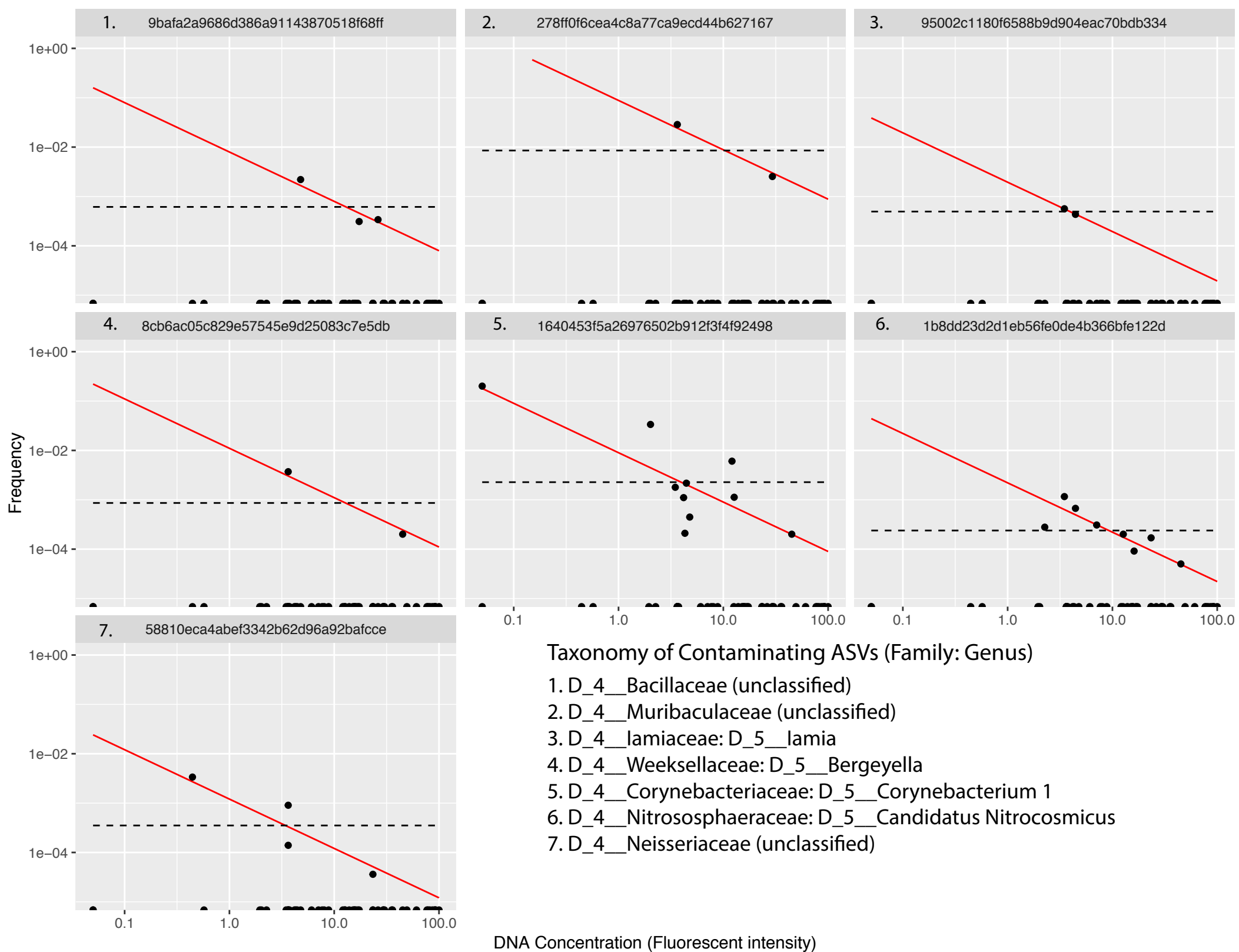

Supplement: Supplementary file 1 [file ECE3-9-6508-s001.pdf]
