# Supplementary material for: Habitat fragmentation is associated with dietary shifts and microbiota variability in common vampire bats
Source: Ecol Evol. 2019 May 9;9(11):6508–23. doi: 10.1002/ece3.5228 (PMC6580296; doi:10.1002/ece3.5228)
Supplement: Supplementary file 2 [file ECE3-9-6508-s002.pdf]

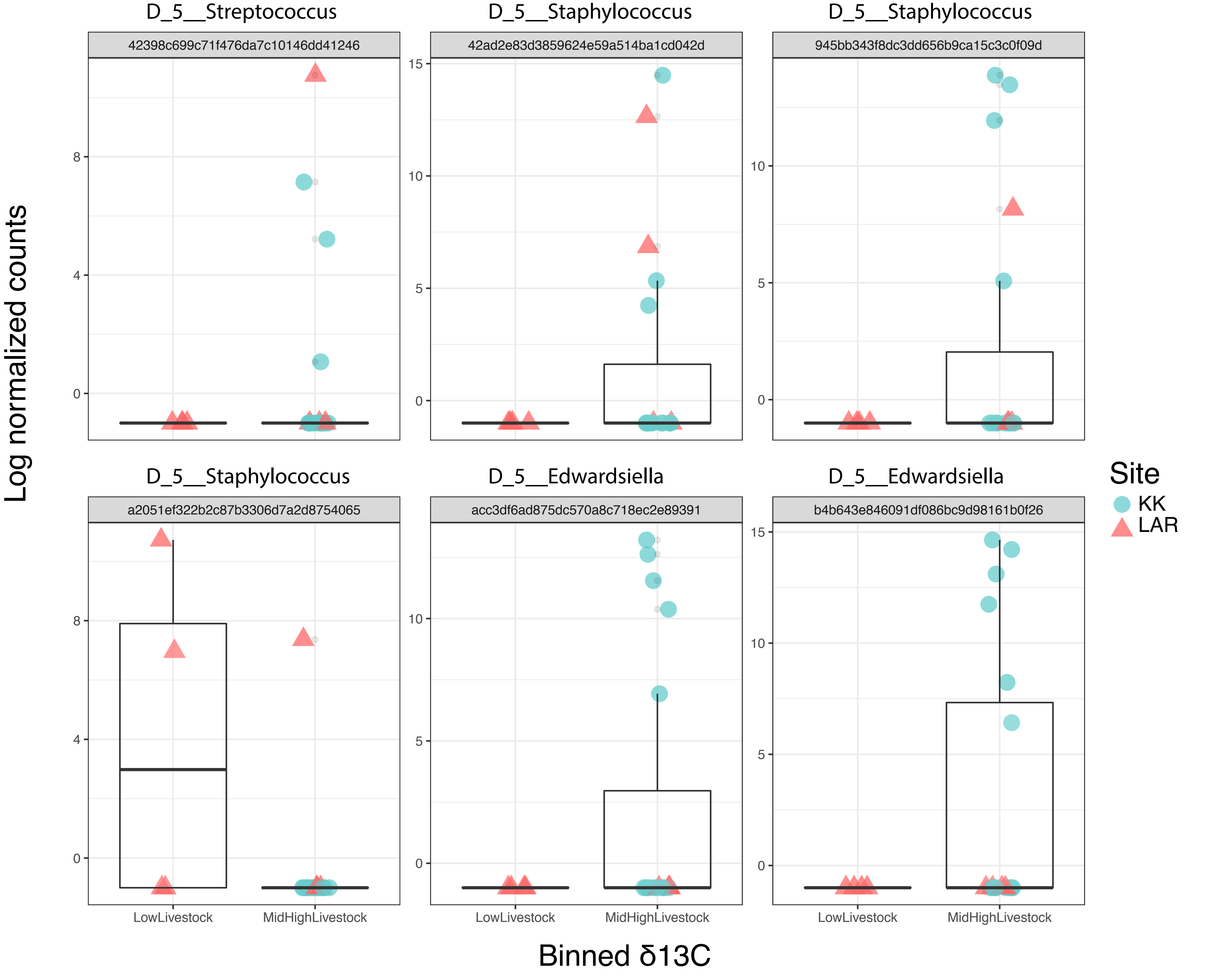

D_5__Streptococcus
42398c699c71f476da7c10146dd41246
D_5__Staphylococcus
42ad2e83d3859624e59a514ba1cd042d
D_5__Staphylococcus
945bb343f8dc3dd656b9ca15c3c0f09d
D_5__Staphylococcus
a2051ef322b2c87b3306d7a2d8754065
D_5__Edwardsiella
acc3df6ad875dc570a8c718ec2e89391
D_5__Edwardsiella
b4b643e846091df086bc9d98161b0f26
Log normalized counts
Binned δ13C
LowLivestock
MidHighLivestock
Site
KK
LAR
